# Timing resolution in double-sided silicon photon-counting computed tomography detectors

**DOI:** 10.1117/1.JMI.10.2.023502

**Published:** 2023-03-23

**Authors:** Christel Sundberg, Mats Persson, J. Jacob Wikner, Mats Danielsson

**Affiliations:** a KTH Royal Institute of Technology, Department of Physics, Stockholm, Sweden; b Prismatic Sensors, Part of GE Healthcare, AlbaNova University Center, Stockholm, Sweden; c MedTechLabs, BioClinicum, Karolinska University Hospital, Solna, Sweden; d Linköping University, Department of Electrical Engineering, Linköping, Sweden

**Keywords:** timing resolution, silicon detector, computed tomography, photon-counting, coincidence detection

## Abstract

**Purpose:**

Our purpose is to investigate the timing resolution in edge-on silicon strip detectors for photon-counting spectral computed tomography. Today, the timing for detection of individual x-rays is not measured, but in the future, timing information can be valuable to accurately reconstruct the interactions caused by each primary photon.

**Approach:**

We assume a pixel size of $12\times 500\;\mu m^{2}$ and a detector with double-sided readout with low-noise CMOS electronics for pulse processing for every pixel on each side. Due to the electrode width in relation to the wafer thickness, the induced current signals are largely dominated by charge movement close to the collecting electrodes. By employing double-sided readout electrodes, at least two signals are generated for each interaction. By comparing the timing of the induced current pulses, the time of the interaction can be determined and used to identify interactions that originate from the same incident photon. Using a Monte Carlo simulation of photon interactions in combination with a charge transport model, we evaluate the performance of estimating the time of the interaction for different interaction positions.

**Results:**

Our simulations indicate that a time resolution of 1 ns can be achieved with a noise level of 0.5 keV. In a detector with no electronic noise, the corresponding time resolution is ∼0.1  ns.

**Conclusions:**

Time resolution in edge-on silicon strip CT detectors can potentially be used to increase the signal-to-noise-ratio and energy resolution by helping in identifying Compton scattered photons in the detector.

## 1 Introduction

Edge-on silicon strip detectors (deep silicon detectors) are emerging as one alternative for photon-counting spectral computed tomography (CT) and currently, prototype detectors are in clinical evaluation.^1^^,^^2^ Compared to conventional CT, in which energy-integrating detectors are used, photon-counting spectral CT uses detectors of semiconductor materials, which allow each interacting photon to be registered based on an electronic pulse that is proportional to the deposited energy. By utilizing energy thresholds, individual photons can be counted and registered with respect to the interaction energy. With photon-counting spectral CT, the energy and spatial resolution can be improved, along with the signal-to-noise ratio (SNR) in the image. The increased spectral information will improve material basis decomposition.^3^

Compared to cadmium-based alternatives, silicon detectors benefit from a mature manufacturing process and can be produced with a high purity and crystalline perfection. Silicon is also compatible with state-of-the art electronic assembly techniques and CMOS electronics can be integrated in the material itself. In comparison to higher Z detectors, silicon has a low probability of K-fluorescence that improves the spectral and spatial resolutions. The silicon is made deep in the direction of the incident x-rays in order to absorb the incoming x-rays efficiently. The detectors can, therefore, be divided into multiple strata in the direction of the incident x-rays, where each stratum only measures a count-rate corresponding to a fraction of the incident photon flux that overall results in a high count-rate capability.

**Fig. 1:**
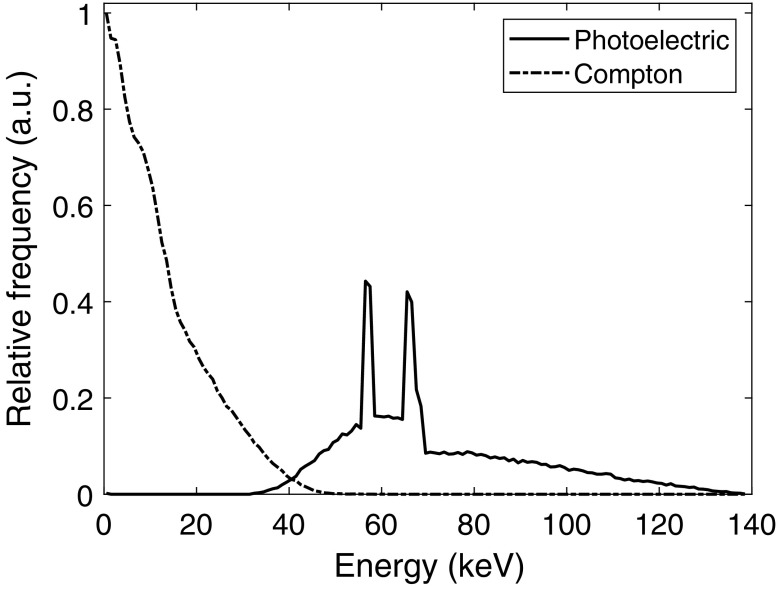
Spectrum of the deposited energies in a silicon detector corresponding to the energies deposited in primary Compton and photoelectric interactions. The spectrum was simulated based on an x-ray source operated at 140 kVp including 8.48 mm aluminum, 0.8 mm beryllium, and 300 mm soft tissue filtration between the source and the detector. A target material of tungsten at an 8-deg angle was assumed and the IPEM Report 78 on x-ray spectral data was used to obtain the incident x-ray spectrum.^4^ For the attenuating materials, the attenuation coefficients were obtained from NIST.^5^ The detector response was simulated in PENELOPE^6^ based on a 4-cm deep silicon detector in the direction of the incident photons.

In silicon detectors, a considerable fraction of the incident photons become Compton scattered in the detector and thereby only deposit some of their energy. In Fig. 1, we show the Compton part and the photoelectric part of the spectrum of deposited energies in a silicon detector. Due to the small overlap in energy, Compton interactions do not contaminate the energy information of photoelectric counts. Further, tungsten shielding is typically used to absorb Compton scattered photons, ensuring that each registered count corresponds to a unique incident photon. Typically, this tungsten shielding also serves as a grid to remove object scatter and thus offsets the loss of geometrical efficiency due to the tungsten. Although the registered energy of each Compton interaction does not correspond to the incident photon energy, the energy information can be utilized in a calibrated detector system and has been shown to improve image quality.^7^ However, it would be desirable to further improve the spectral performance, for example, by distinguishing all interactions induced by each incident photon.

The probability of Compton scattering decreases with a decreasing photon energy. In a detector with no tungsten shielding, it is possible for incident photons to Compton scatter multiple times in the detector. If such a chain of interactions ends in a photoelectric event, the entire photon energy will be deposited in the detector. By identifying interactions that originate from the same incident photon, it is possible to obtain the incident photon energy by adding the registered interaction energies together. If the interaction chains are correctly identified, this will have a direct impact on the detector performance. From a photon-counting perspective, the number of photons that become double-counted due to Compton scattering will be reduced. Further, the spectral performance will increase since the photon energy can be estimated based on the interaction energies. We have previously presented a method to estimate the incident photon energy in a detector with no tungsten shielding using a maximum-likelihood approach.^8^ For incident photons of 60 keV, it was shown that, given a set of interactions originating from the same incident photon, the photon energy could be estimated with an accuracy of a few keV. However, due to the high incident photon flux associated with CT, isolating interactions that originate from the same incident photon is difficult. To mitigate this, it is desirable to decrease the number of incident photons per sampling period. This can be done geometrically, by introducing tungsten shielding in order to limit the area where coincidences can occur, and using a detector with high timing resolution, thereby decreasing the sampling period.

One way of achieving high timing resolution is by using a detector with double-sided readout electrodes. When a photon interacts in a semiconductor detector, energy is deposited which results in released electron-hole pairs. Due to an applied bias voltage, there is an electric field across the detector that causes holes to drift to one side of the detector and electrons to drift in the opposite direction. The movement of charge carriers induces currents on the collecting electrodes. In a detector with double-sided readout, electrodes are located on both sides of the detector. For each interaction, depending on the size of the electrodes in relation to the charge clouds, there will be at least two induced current signals, one from each side of the detector.

The appearance of the induced current pulse is governed by the electrode size and position in relation to the neighboring electrodes. If very small electrodes are used, the induced currents will be dominated by charge movement close to the electrodes. Since holes and electrons are collected on different sides of the detector, the signals generated on each electrode largely arise due to the movement of the charge carrier type that is collected by the electrode. Typically, the charge carriers arrive at the electrodes within a short time window which results in induced current signals that have distinct peaks and are large for only a short period of time. We have previously presented a simulation study of a single-sided silicon detector with a pixel size of $12\times 500\;\mu m^{2}$ that will exhibit these features.^9^

Depending on the interaction position, the drift path to the collecting electrodes varies for the released charge carriers. This, in combination with the difference in mobility, can lead to electrons and holes arriving at the collecting electrodes on each side of the detector at different times. In the case of small electrodes, since the induced signals consist of sharp peaks, it is possible to compare the timing of the signals between the front-side and the back-side electrodes in order to estimate the time of the interaction.

By estimating the time of the interaction, it is also possible to infer the interaction position based on the known charge carrier mobilities. Using double-sided detectors to improve the spatial resolution has previously been evaluated, aiming for applications in mammography.^10^ In general, the concept of silicon detectors with double-sided strips for position-sensitive readout is well-known and detectors have been presented for various applications, e.g., in space science, particle physics, Compton telescopes, positron emission tomography (PET), and single positron emission tomography (SPECT).^11^^–^^16^ One common detector configuration is to have orthogonal strips on opposing surfaces of the detector and thereby infer the interaction position in two dimensions by correlating the measured signals from each side of the detector.^17^ Further, by including timing information of the pulses, the interaction position can be determined in the third dimension.

In this work, we present a simulation study, in which the time of interaction is estimated based on a double-sided deep silicon detector for CT with parallel facing electrodes and a pixel size of $12\times 500\;\mu m^{2}$. For this, photon interactions were Monte Carlo simulated and a charge transport model was applied to study the induced current pulses as functions of the interaction position. Based on the induced currents resulting from each interaction, the time of each induced pulse was estimated and used to infer the time of the interaction. We also include a realistic model of electronic noise for which we evaluate the performance of estimating the time of interaction.

## 2 Method

### 2.1 Detector Simulations

**Fig. 2:**
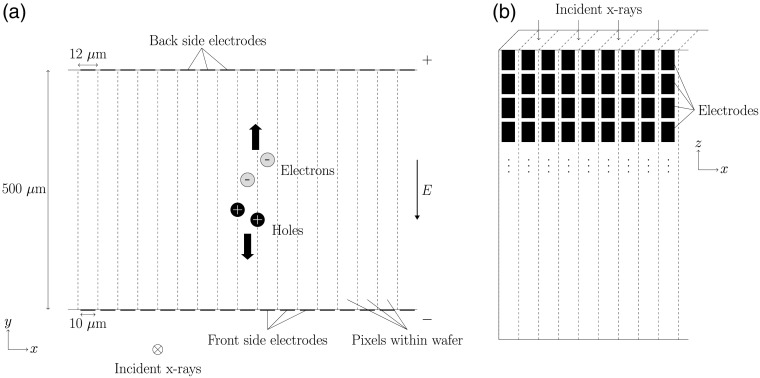
(a) Simulated detector geometry as seen from the perspective of the x-ray source. The detector consists of a 500-μm thick silicon wafer that is divided into virtual pixels by electrodes located on each side of the detector. Each electrode is 10μm wide and the pixel pitch is 12  μm. The electrodes on both sides of the wafer have the same geometry and orientation. The incident x-rays enter the detector in the negative z direction. An example of released charge carriers is also included. The large black arrows indicate the drift direction of the electrons and holes, respectively. (b) The assumed detector has several separate rows of electrodes in the direction of the incident x-rays. In this work, the length of the electrodes in this direction was not defined and all simulations were performed in the xy-plane, assuming that the electrode size in the z direction is large in relation to the electrode size in the x direction.

To obtain the induced current signals, an edge-on silicon detector with double-sided readout was simulated. For this, we assumed a detector geometry in which strip electrodes are located on two sides of the silicon wafer. The electrodes on both sides of the wafer were assumed to have the same dimensions and orientation, meaning that each electrode on one side of the silicon wafer has a counterpart electrode on the other side of the wafer, i.e., the alignment of the electrodes is not orthogonal on opposing sides of the detector. A wafer thickness of 500  μm was assumed along with an electrode width of 10  μm and a pixel pitch of 12  μm. This corresponds to a virtual pixel area of $12\times 500\;\mu m^{2}$ facing the x-ray source. A drawing of the detector geometry can be seen in Fig. 2.

**Fig. 3:**
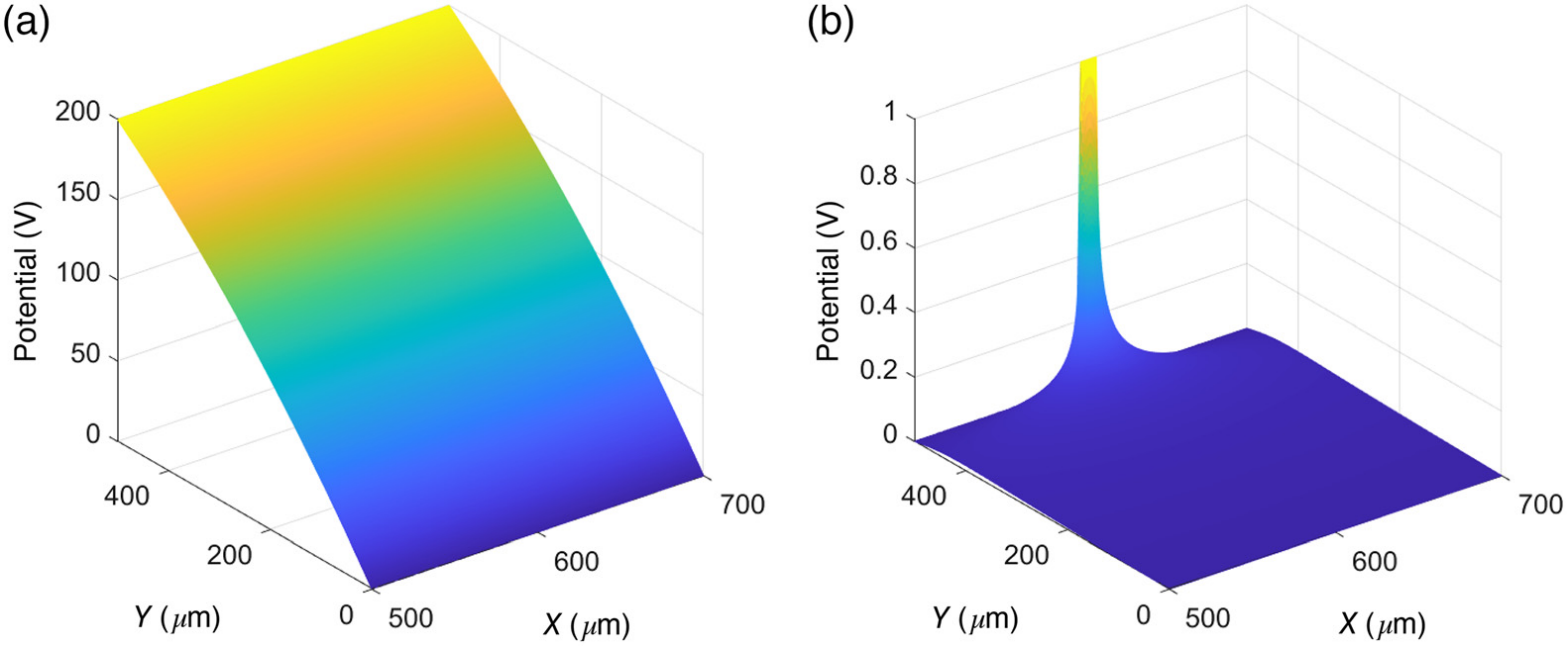
(a) Electric potential based on a simulated detector with a bias voltage of 200 V applied to the back-side electrodes, here located at Y=500  μm. (b) Weighting potential of the investigated pixel at the back side of the detector based on a 10-μm electrode width and 12-μm pixel pitch. The potential is 1 V at the position of the electrode. For the front-side electrode, the weighting potential is obtained by rotating the presented distribution 180 deg, so that the electrode is located at a y position of 0  μm.

To obtain the electric field, the detector was realized as a silicon wafer with 200 pixels. The detector was assumed to be reverse biased with 200 V applied to the back-side electrodes. Based on this, the electric potential was calculated numerically by solving the Poisson equation $\nabla^{2}\psi =-\frac{qN}{\epsilon }$, where q is the elementary charge, ε is the silicon permittivity, N is the acceptor concentration, and ψ is the electric potential. The acceptor concentration N was calculated as $N=\frac{1}{q\rho \mu_{m}}$, where ρ is the resistivity and $\mu_{m}$ is the mobility of the majority carrier. Throughout this work, a silicon resistivity of 15  kΩ·cm was assumed. The electric potential was set to be equal to the bias voltage on the back-side electrodes and zero on the front-side electrodes. The resulting electric potential is presented in Fig. 3(a).

The initial charge distribution resulting from each photon interaction, the electron track, was simulated in PENELOPE, a Monte Carlo simulation package that simulates coupled electron–photon showers in the range 50 eV to 100 MeV.^6^ For this, photon interactions of 10 and 70 keV were simulated. Based on the spectrum of deposited energies in a silicon detector, from an x-ray source operated at 140 kVp including 30 cm soft tissue filtration (Fig. 1), 10 and 70 keV approximately correspond to the average energy of the Compton part and the photoelectric part of the spectrum, respectively. The interactions were simulated in nine different positions in the y direction according to 10%, 20%, …, 90% of the wafer thickness as measured from the front-side electrode. In the x direction, interactions were either centered on the electrodes (located at the pixel middle) or positioned at the pixel edge. In total, 1000 interactions were simulated for each interaction position. Based on the simulated charge distribution given by PENELOPE, electron–hole pairs were created by dividing the energy deposited in each position by the silicon ionization energy of 3.6 eV.^18^ In this work, only primary interactions were considered since we expect any secondary interactions to occur in different pixels compared to the primary interactions and therefore not affect the induced current signals that result from the primary interaction.

In this work, charge transport was considered from a two-dimensional perspective. This represents a case in which the electrodes are assumed to be long in the z direction meaning that we expect no nonuniformities along that direction. Due to the applied electric field, the released electron–hole pairs are separated immediately after generation. The charge carriers then drift to each side of the detector with a drift velocity, $\bar{v_{d}}$ that is dependent on the charge carrier mobility μ and the electric field $\overset{¯}{E}$ according to $\bar{v_{d}}=\mu \bar{E}$. In this work, field-dependent mobilities were used to calculate the drift velocity for holes and electrons, respectively.^19^

Diffusion causes the drifting charge clouds of electrons and holes to increase in size. In this work, for each time step, the diffusion distance dx in the x direction was sampled according to a Gaussian distribution P(x) expressed as

$$P(x)=\frac{1}{\sqrt{4\pi Ddt}}\;\exp(-\frac{x^{2}}{4Ddt}),$$ (1)

where D is the diffusion coefficient given by the Einstein law $D=\frac{kT}{e}\mu$, where k is the Boltzmann constant, T is the absolute temperature, and e is the fundamental electron charge. The time step dt was set to 0.05 ns in order to achieve sufficient detail in the induced current pulse shapes. The diffusion distance in the y direction, dy was sampled according to the corresponding distribution P(y).

The total velocity $\overset{¯}{v}$, including drift and diffusion, is then given by

$$\bar{v}=\bar{v_{d}}+\bar{v_{D}},$$ (2)

where $\bar{v_{D}}$ describes the velocity caused by diffusion calculated as $\bar{v_{D}}=(dx/dt,dy/dt)$. It should be noted that Coulomb repulsion is currently not included in our simulation framework.

The charge movement due to drift and diffusion results in induced currents on the electrodes on each side of the detector. The induced current on each electrode is given by the Shockley–Ramo theorem:^20^^,^^21^

$$i(t)=e{\bar{E}}_{w}(x,y)\bar{v}(t),$$ (3)

where i(t) is the induced current, ${\bar{E}}_{w}(x,y)$ is the weighting field for a charge carrier in position (x,y), $\bar{v}$ is the carrier velocity, and t is the drift time. For each electrode, the weighting potential W was obtained by solving the Laplace equation in two dimensions with the electrode set to unit potential and all other electrodes at ground potential. From this, the weighting field was obtained as ${\bar{E}}_{w}(x,y)=-\nabla W$. In Fig. 3(b), the weighting field for an electrode located at the back side of the detector is presented. With the detector geometry presented in this work, holes will be collected by the front-side electrodes and electrons by the back-side electrodes. Throughout this work, a detector material of high purity was assumed and trapping effects were therefore not considered.

### 2.2 Readout Electronics

In a realistic detector implementation, it is not possible to extract the induced currents directly. Instead, readout electronics are required to amplify and process the signals. In photon-counting spectral CT, for example, the induced current pulse is processed such that the resulting pulse has a pulse height that corresponds to the photon energy, which can then be quantified using energy thresholds. The processing typically starts with an integration stage, a charge sensitive amplifier (CSA), that accumulates the charge and represents the induced current with a voltage of more significant magnitude. However, any additional circuitry adds electronic noise to the signal. To increase the SNR, the noise is attenuated through filtering and signal pulse shaping.

**Fig. 4:**
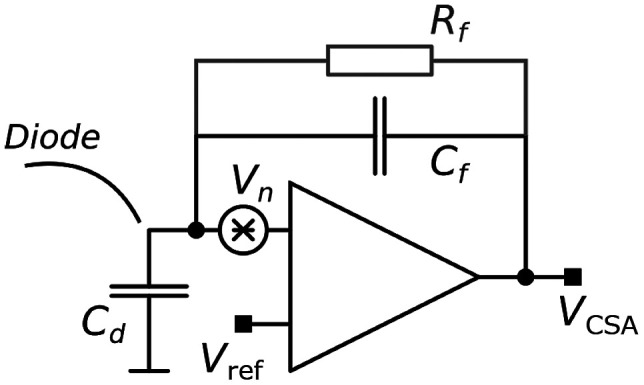
Illustration of the CSA at the input of the readout electronics.

In this work, the time of each interaction was estimated by performing a least-square fit to the processed induced current signals. It is possible to perform this fit by extracting the signal at several different places in the readout chain. By extracting the signal before it is filtered, the electronic noise can be assumed to be white, which simplifies the least-square fitting. Given that an optimal estimator is found, this will give the same results as extracting the signal after filtering.^22^ However, since filtering introduces correlated noise, extracting the signal before this stage will simplify the implementation of the estimator. The CSA is a given component in the readout electronics of any photon-counting detector. Theoretically, it is a linear operator that integrates the induced current pulse from the diode. The noise from the diode is averaged over the pulse duration and in general, the CSA provides robustness against signal fluctuations, dark current, and noise. In this work, we have therefore extracted the signal directly after the CSA. To model this, we use an idealized model of the electrical interface in order to not obscure the scope of this paper by introducing too many other design parameters. In Fig. 4, the front-end CSA is illustrated assuming a diode-related capacitance $C_{d}$, a feedback (integrating) capacitance $C_{f}$, and a feedback resistance $R_{f}$ for recovery.

**Fig. 5:**
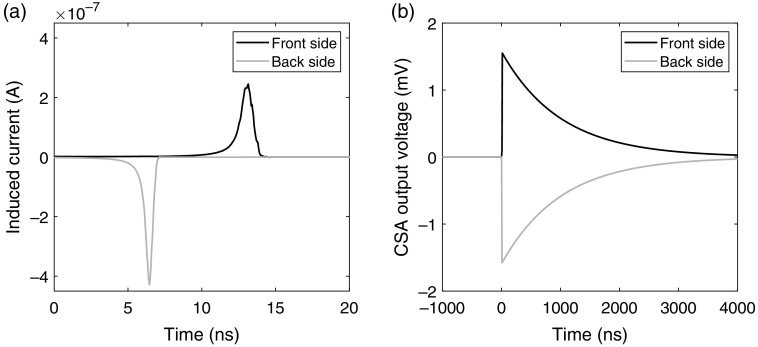
(a) Simulated induced current signal from an interaction of 10 keV at the center of the pixel in both the x and y directions, i.e., the distance to the front-side electrode is equal to the distance to the back-side electrode. (b) CSA output voltage based on the induced current pulses. The CSA accumulates the charge and represents the induced current with a voltage of more significant magnitude.

In the frequency domain, the output voltage from the CSA, $V_{\mathrm{CSA}}(s)$, is given by

$$V_{\mathrm{CSA}}(s)=\frac{T}{C_{f}}\frac{1}{\left(1+sT\right)}I_{\text{signal}}(s),$$ (4)

where $T=C_{f}R_{f}=1\;\mu s$ is a time constant that describes the relaxation of the CSA, s=jω is the complex angular frequency (assuming a stable system), $C_{f}=200\;\mathrm{fF}$ is the feedback integration capacitance of the CSA, and $I_{\text{signal}}(s)$ is the induced current signal.^23^ In the time domain, an example of the induced current signals on the front-side and back-side electrodes is presented in Fig. 5 along with the corresponding CSA output signals. The simulated pulses correspond to an interaction in the middle of the pixel in the x direction (along the wafer thickness) and the y direction (across the wafer thickness).

**Fig. 6:**
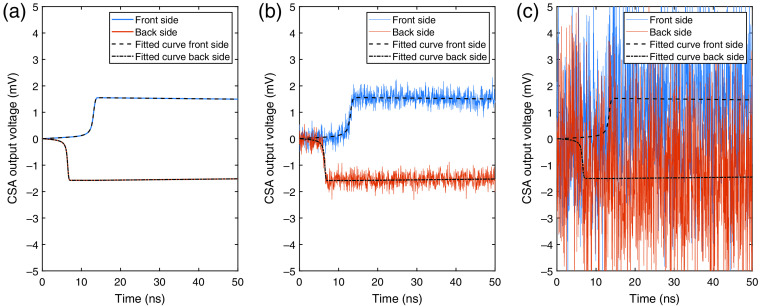
CSA output voltage along with fitted template curves for the three noise cases based on an interaction of 10 keV. The input currents correspond to an interaction at the pixel center: i.e., equidistant to the electrodes and to the pixel edges in the x direction. (a) Ideal detector with no noise. (b) Noise corresponding to a standard deviation of σ=0.05  keV. (c) Noise corresponding to a standard deviation of σ=0.5  keV. For the two noise cases, it should be noted that the noise levels in keV are given according to the size at the output of the entire readout channel (including filtering) and not at the CSA output.

#### 2.2.1 Simulated noise

In silicon detectors, it is desirable to have low electronic noise to detect Compton interactions, which range down to 0 keV. Typically, based on the noise level, a lowest energy threshold is set to minimize the number of undesired noise counts while still counting as many Compton interactions as possible. This threshold is normally applied to the signal after filtering and pulse shaping.

The dominating noise in the simulated readout chain is modeled as voltage fluctuations on the CSA input gate as we assume CMOS technology. Further on, noise from the feedback resistance and the diode are omitted from this analysis as we assume an idealized model of the electrical interface. Assuming that the virtual node (indicated by the diode pointer) is $V_{x}$, we have according to Kirchoff’s current law

$$(0-V_{x})sC_{d}+(V_{\mathrm{CSA}}-V_{x})Y_{f}=0\Rightarrow \frac{V_{\mathrm{CSA}}}{V_{x}}=\frac{sC_{d}+Y_{f}}{Y_{f}},$$ (5)

where $Y_{f}=(1+sC_{f}R_{f})/R_{f}=(1+sT)/R_{f}$.

In a small-signal analysis, we can take the noise into account and equal the virtual ground to the fluctuations on the CMOS gate as $V_{x}=V_{n}$. The PSD $S_{\mathrm{CSA}}$ at the CSA output thus becomes

$$S_{\mathrm{CSA}}=V_{n}^{2}{\left|\frac{sC_{d}+Y_{f}}{Y_{f}}\right|}^{2}=V_{n}^{2}{\left|\frac{1+s(C_{d}+C_{f})R_{f}}{1+sT}\right|}^{2},$$ (6)

where $V_{n}^{2}$ is the noise PSD associated with the input CMOS gate and is assumed to be white. In a reasonable implementation, $R_{f}$ will have a large value. This results in a PSD according to

$$S_{\mathrm{CSA}}\approx V_{n}^{2}{\left|\frac{C_{d}+C_{f}}{C_{f}}\right|}^{2},$$ (7)

which is a constant that only scales the white input noise, making the resulting output noise white as well. Even if $R_{f}$ is of moderate size, the expression will have a pole-zero pair which are fairly close in frequency. The diode capacitance $C_{d}$ is assumed to be small, since we are considering very small pixels. Since $C_{d}\approx C_{f}$, the noise PSD scales the input white noise from a factor 1 at low frequencies to a factor 2 at high frequencies. In this work, we assume that this slightly colored noise has a negligible impact on the overall discussion.

To simulate the electronic noise, Gaussian white noise was added to the CSA output signals resulting from the simulated induced currents on each electrode. It was assumed that the noise between the electrodes is uncorrelated. In order to achieve a desired noise level, the input noise was scaled based on the SNR at the output of the entire readout chain, including filter and pulse shaping. For this, we assumed a previously presented design of the front-end electronics.^24^ With S(s) as the signal at the CSA output, the output signal from the entire readout chain can be approximated as

$$S_{\text{entire}}(s)=\frac{C_{\mathrm{pz}}}{C_{s}T}\frac{\tau_{s}(1+sT)}{\left(1+s\tau_{s}\right)}{\left(\frac{2}{1+s\tau_{0}}\right)}^{2}S(s),$$ (8)

where $C_{\mathrm{pz}}=1600\;\mathrm{fF}$ is the capacitance of the pole-zero cancellation circuit, $C_{s}=200\;\mathrm{fF}$ is the shaper amplifier capacitance s=jω, where j is the imaginary unit and ω is the angular frequency, and $\tau_{s}=\tau_{0}=20\;\mathrm{ns}$ are the time constants associated with the shaper amplifier and the filter, respectively.

With no added electronic noise, i.e., $S(s)=V_{\mathrm{CSA}}(s)$, the output from the readout chain is a pulse with a peak amplitude that is proportional to the energy deposited by the interacting photon. From the pulse peak, it is possible to observe the gain of the readout chain which translates voltage to energy in keV. Based on the determined gain, the input noise at the CSA can be scaled so that the desired SNR at the output of the readout chain is achieved. The output noise power is typically calculated as the standard deviation of the noise signal expressed in keV rms.

In this work, the performance of estimating the time of the interaction was evaluated for three different noise cases. First, assuming an ideal detector with no electronic noise. Then for a potential future detector with very low electronic noise, a noise level corresponding to a standard deviation of σ=0.05  keV at the output from the entire readout chain. Finally, based on a realistic detector, a noise level corresponding to σ=0.5  keV.

In an implementation, it is reasonable to assume that a majority of the noise comes from the integrator, but we also have to allocate some of the noise to the filter after the integrator. In this work, we assume that 80% of the noise originates form the integrator based on good-practice design of the electronic devices. For example in the case of σ=0.5  keV, this means that we assume that 0.5·0.8=0.4  keV originates from the integrator. In this work, we refer to the noise level as measured at the output from the entire readout chain. However, it should be noted that the noise level in relation to the signal amplitude will be higher at the CSA output since the signal is unfiltered at this stage.

Throughout this work, the simulated readout electronics was implemented in MATLAB (The Mathworks Inc., Natick, Massachusetts, United States) based on Eqs. (4) and (8). The induced current signals were Fourier transformed by applying the discrete Fourier transform and propagated to the output of the CSA based on Eq. (4). The noise at the CSA output was realized in the frequency domain as Gaussian white noise with zero mean and standard deviations corresponding to the desired noise levels of σ=0.05  keV and σ=0.5  keV at the output of the readout chain. To obtain the total output of the entire readout chain (signal and noise), the CSA output signal and the simulated noise were added and propagated based on Eq. (8). The time-domain output signals at the CSA output and at the output of the entire readout chain were obtained using the inverse Fourier transform. Throughout this work, a sampling rate of 20  GS/s was assumed in order to resolve the induced current pulses sufficiently.

### 2.3 Time of Arrival Estimation

Based on the simulated detector geometry with electrodes on two opposite sides of the wafer, each interaction will result in at least two induced current pulses. Due to the small electrode size in relation to the wafer thickness, the weighting field will be large only very close to the electrodes. This results in induced current signals that are dominated by charge movement close to the electrodes. Because of this, the induced current pulses have narrow peaks and are large only for a short period of time. Depending on the interaction position, the charge carriers will arrive at each side of the detector at different times. Since the induced current pulses occur from charge movement in the close proximity of each electrode, the induced current pulses provide an estimate of the time of the charge collection. By comparing the time of the charge collection between at least two opposing electrodes, it is possible to estimate the time of the interaction.

To achieve this, in this work, the simulated currents from the two opposing electrodes corresponding to the illuminated pixel were extracted. These signals were then processed through the described readout electronics. Based on the 1000 realizations for each interaction position, a template curve was calculated for each of the electrodes based on the average of the CSA output pulses. For this, no noise was included in the simulated readout electronics.

For each interaction position, the time of the front-side pulse and the back-side pulse resulting from each interaction was estimated by fitting the template curves to the CSA output signal at the front-side and back-side electrodes, respectively. The fitting was performed by minimizing the error between each realization of the signal and the template curve corresponding to the same interaction position and electrode. In the optimization, three parameters were fitted: the amplitude of the template curve, the time step of the template curve, and the position of the template curve in time. The time step then governs the slope of the fitted curve and corresponds to differences in pulse width between the template curve and each simulated induced current pulse. To find the minimum mean square error, the MATLAB function fminsearch was used. For each electrode, based on the fitted template pulses, the time of the half maximum was extracted and used as the time estimate of the pulse.

In the optimization, each realization was extracted as a 100-ns long signal. The time of each interaction was chosen randomly within the first 5 ns of the 100-ns time window. The template curves were extracted on a larger time interval in order to ensure a good fit. For this, each template signal was extracted as a 1000-ns long pulse with the signal occurring at time 50 ns. The curve fitting was performed separately for all three noise cases: ideal detector with no added noise, noise corresponding to σ=0.05  keV, and noise corresponding to σ=0.5  keV. For the cases with a nonzero noise level, different realizations of added noise were used for each set of interactions. In Fig. 6, examples of the CSA output signals are shown for all three noise cases along with the fitted template curves that were used to estimate the time of the interaction.

**Fig. 7:**
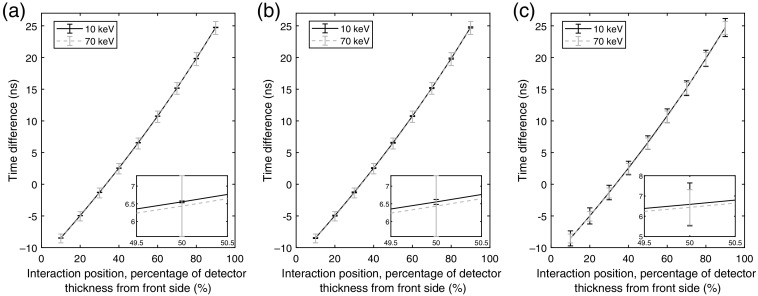
Average time differences between the time estimates of the front-side and back-side induced pulses for interactions of 10 and 70 keV. The positions indicate the interaction position in the y direction and are presented as percentages of the wafer thickness from the front-side electrode. In the x direction, all interactions are located in the pixel middle, i.e., centered on the electrodes. The included error bars indicate the standard deviation of the estimated time differences for each position. The average time differences are based on 1000 realizations for each interaction position and are presented for each of the assumed noise cases. (a, d) Ideal detector with no noise. Noise corresponding to a standard deviation of (b, e) σ=0.05  keV and (c, f) σ=0.5  keV.

Based on the resulting time estimates for each interaction, the time difference between the front-side pulse and the back-side pulse was calculated. The time difference was first evaluated with respect to the interaction position to give an indication of the performance with respect to spatial resolution. The time difference was then evaluated with respect to the time of the interaction. For this, we introduce the charge collection time $T_{c}$, which describes the average time between the interaction and the pulses at the front-side and back-side electrodes. This is written as

$$T_{c}=\frac{\left(t_{\text{front}}-t_{\text{interaction}})+(t_{\text{back}}-t_{\text{interaction}}\right)}{2},$$ (9)

where $t_{\text{front}}$ is the estimated time of the front-side pulse, $t_{\text{interaction}}$ is the actual time of the interaction, as known from the simulation, and $t_{\text{back}}$ is the estimated time of the back-side pulse.

From the simulated data, a general expression for the charge collection time $T_{c,\text{fit}}(t_{\text{front}}-t_{\text{back}})$ was obtained by fitting a curve to the charge collection time for each interaction as a function of the time difference between the front-side and back-side pulses. This was performed separately for each of the two simulated interaction energies (10 and 70 keV) and for the three noise cases. For each energy and noise case, separate curves were fitted, first to the data corresponding to interactions in the pixel middle, then for the data corresponding to interactions on the pixel edge, and last for the combined dataset based on both interactions in the pixel middle and on the pixel edge. The resulting timing resolution was then calculated as the standard deviation of the errors between the fitted curve and the simulated data.

With each fitted curve $T_{c,\text{fit}}(t_{\text{front}}-t_{\text{back}})$, the time of the interaction is then given by

$$t_{\text{interaction}}=t_{\text{back}}+\frac{t_{\text{front}}-t_{\text{back}}}{2}-T_{c,\text{fit}}(t_{\text{front}}-t_{\text{back}}).$$ (10)

## 3 Results

Based on the time estimates obtained from the fitted curves, the resulting mean time difference between pulses at the front side and back side of the detector is presented as a function of the interaction position in the y direction in Fig. 7. In this figure, the interaction positions in the y direction are indicated as percentages of the wafer thickness, e.g., with 10% being 10% of the wafer thickness from the front-side electrode. The included error bars indicate one standard deviation based on the estimated time differences for each interaction position.

**Fig. 8:**
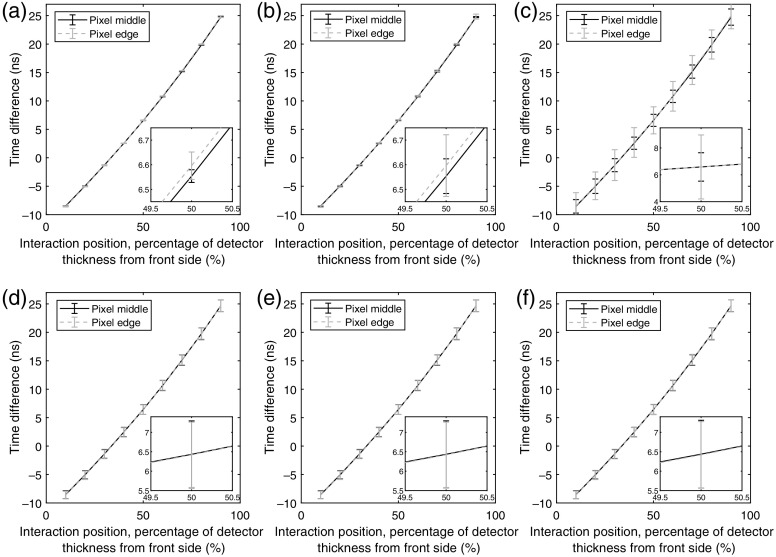
Average time differences between the time estimates of the front side and back-side induced pulses. (a)–(c) The results for interactions of 10 keV and (d)–(f) interactions of 70 keV. The interaction positions are indicated as percentages of the wafer thickness from the front-side electrode. The two curves in each subfigure show results for two different interaction positions in the x direction: the pixel middle and the pixel edge. The pixel middle data points are the same as in Fig. 7. The included error bars indicate the standard deviation of the estimated time differences for each position. The average time differences are based on 1000 realizations for each interaction position and are presented for each of the assumed noise cases. (a, d) Ideal detector with no noise. Noise corresponding to a standard deviation of (b, e) σ=0.05  keV and (c, f) σ=0.5  keV.

**Fig. 9:**
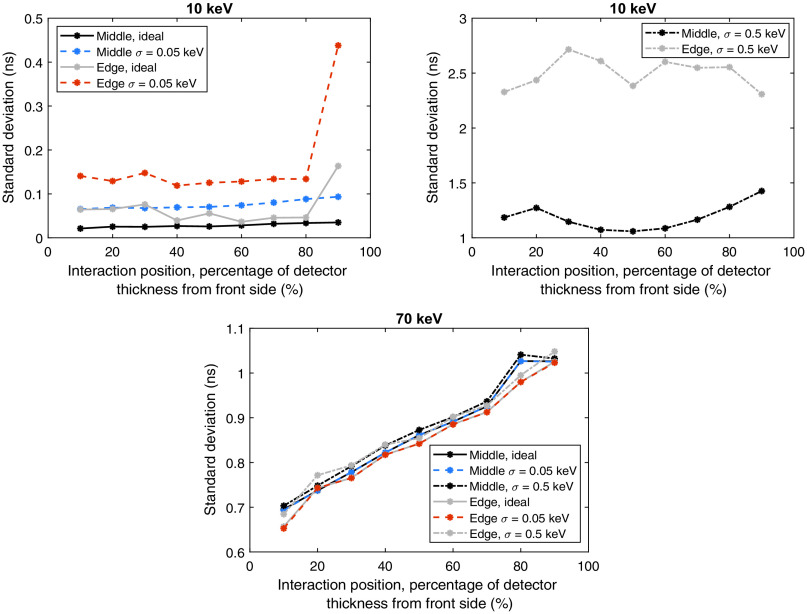
Standard deviations of the time differences between the time estimates of the front-side and back-side induced pulses for interactions of 10 and 70 keV. For each energy, curves are presented for all three noise cases as well as for the two positions in the x direction (pixel middle and pixel edge). Each data point corresponds to the standard deviation of 1000 pulse realizations.

In Fig. 8, the effect of the interaction position in the x direction is evaluated for the 10- and 70-keV cases. Figures 8(a)–8(c) show the results for interactions of 10 keV and Figs. 8(d)–8(f) represent the case with interactions of 70 keV. Similar to Fig. 7, the presented percentages indicate the position of the interaction in the y direction. The standard deviations used in the error bars of Figs. 7 and 8 are presented in Fig. 9 for each interaction position, energy, and noise assumption.

**Fig. 10:**
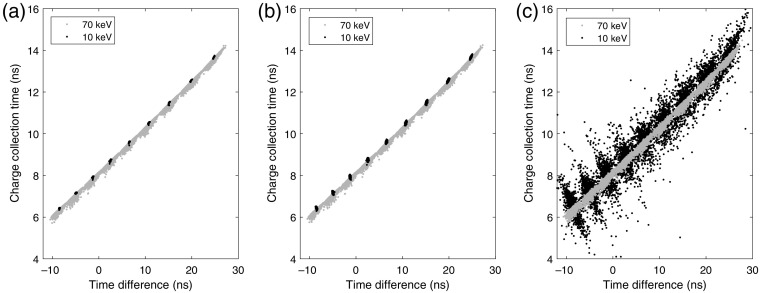
Charge collection time as a function of the time difference between the time estimates of the front-side and back-side pulses for interactions of 10 and 70 keV. The interactions are centered on the electrodes in the x direction. With an interaction at time t=0, the charge collection time describes the time between the interaction and the average time of the front-side and back-side induced pulse. Each scatter plot shows 9000 realizations (1000 realizations for each of the 9 positions between the front-side and back-side electrodes) for each energy and noise case. (a) Ideal detector with no noise. Noise corresponding to a standard deviation of (b) σ=0.05  keV and (c)σ=0.5  keV.

Regarding time resolution, the charge collection time as a function of the time difference between the time estimates of the front-side and back-side pulses is presented in Fig. 10 for interactions of 10 and 70 keV at the pixel middle in the x direction. Figures 10(a)–10(c) show the results for the three noise cases: ideal, σ=0.05  keV, and σ=0.5  keV.

**Fig. 11:**
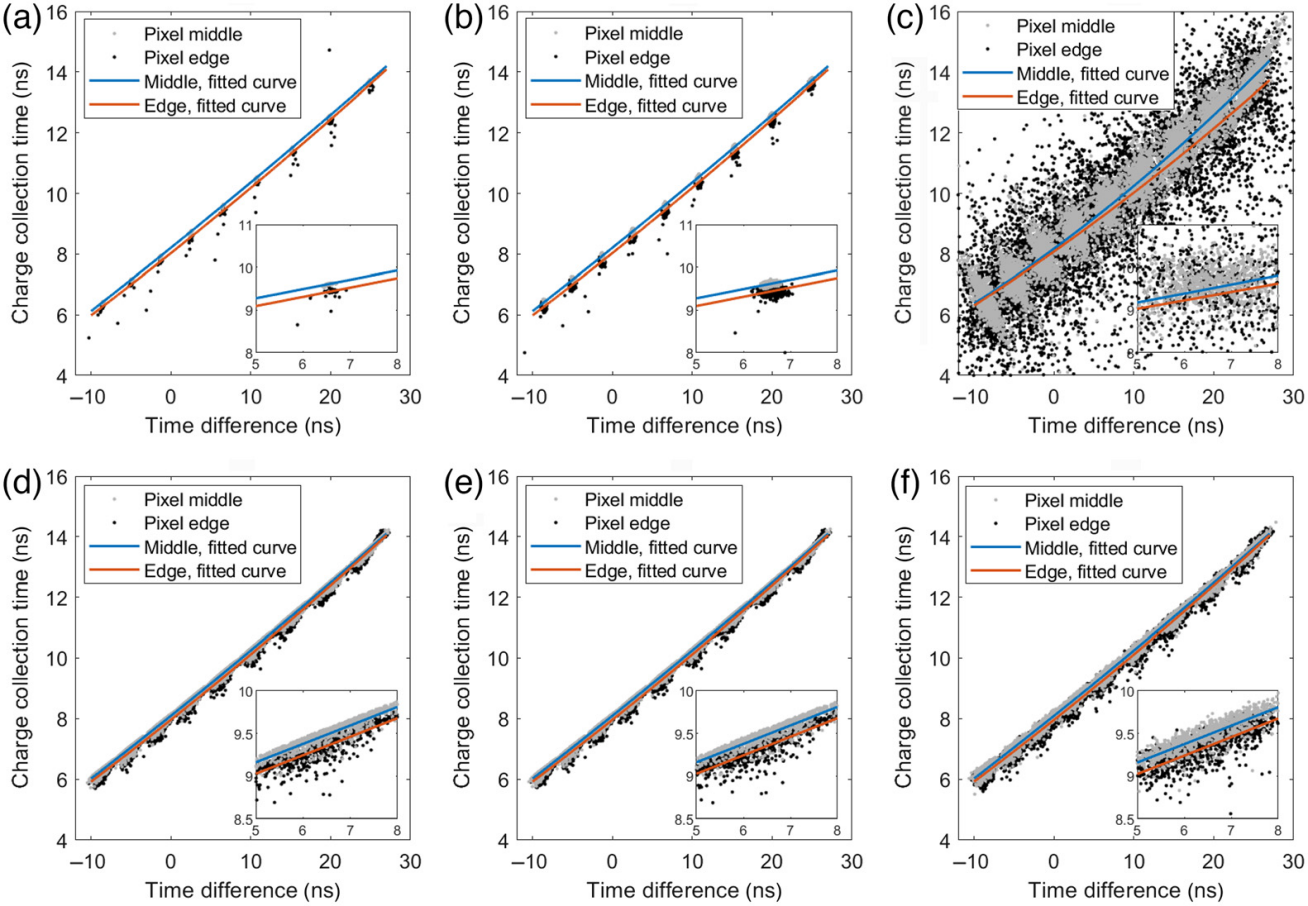
Charge collection time as a function of the time difference between the time estimates of the front-side and back-side pulses for interactions of (a)–(c) 10 keV and (d)–(f) 70 keV. With an interaction at time t=0, the charge collection time describes the time between the interaction and the average time of the front-side and back-side induced pulse. Results are shown for two different interaction positions in the x direction: the pixel middle and the pixel edge. The pixel middle data points are the same as in Fig. 10. Each scatter plot shows 9000 realizations (1000 realizations for each of the 9 positions between the front-side and back-side electrodes) for each energy and noise case. (a, d) Ideal detector with no noise and noise corresponding to a standard deviation of (b, e) σ=0.05  keV and (c, f) σ=0.5  keV. For each position in the x direction, a curve was fitted to the data.

The charge collection time is presented for interaction positions at the pixel middle as well as at the pixel edge in Fig. 11. Figures 11(a)–11(c) show the results for the 10-keV case and Figs. 11(d)–11(f) show the results for the 70-keV case. In this figure, each column represents a noise case and each subfigure includes two second-order polynomial curves that were fitted to the data for each position in the x direction.

In Table 1, the standard deviation of the errors between the fitted curves and the data is shown for each photon energy, interaction position, and noise case. This table also shows the results from two additional curves that were fitted to the combined data from both the pixel edge and the pixel middle for each energy and noise case.

**Table 1:** Standard deviation of the errors between the fitted curves that represent the charge collection time and the corresponding data for each photon energy, interaction position in the x direction, and noise level. The two columns with combined data represent cases for which a curve was fitted to the combined data from both the pixel middle and the pixel edges.

	Standard deviation (ns)					
	10 keV			70 keV		
	Pixel middle	Pixel edge	Combined data	Pixel middle	Pixel edge	Combined data
Ideal	0.01	0.05	0.09	0.07	0.08	0.09
Noise σ=0.05 keV	0.03	0.12	0.12	0.07	0.08	0.09
Noise σ=0.5 keV	0.62	1.49	1.15	0.10	0.13	0.13

## 4 Discussion

From Fig. 7, it can be seen that the average time difference between front-side and back-side induced pulses varies with the interaction position. For positions close to the front side, the front-side pulses occur before the back-side pulses that result in a negative time difference. At slightly more than 30% from the front-side electrode, the time difference becomes zero, which means that the pulses occur at the same time. Overall, the lower mobility of holes results in a time difference that increases with an increasing distance to the front-side electrodes. For all three noise cases, the average time differences are very similar regardless of photon energy. As can be seen in the zoomed subplots, the average time differences for the 70-keV case are slightly lower than for the 10-keV case.

For the ideal case with no noise, the time differences for the 10-keV case show a very small standard deviation, expressed by the included error bars [Fig. 7(a)]. In comparison, the 70-keV case shows a much larger standard deviation. This is caused by the initial distribution of charge carriers. For the 70-keV case, the electron track along which charge carriers are released can be several micrometers, while for 10 keV, the track length is typically less than a single micrometer. The initial track length affects the time required for all charge carriers to be collected. This alters the width of the induced current pulse that is measured on the electrodes. The larger the initial charge distribution, the wider the induced current pulse. Further, with an increasing track length, the difference between the initial interaction position and the effective interaction position, as given by the positions of all released charge carriers, increases. The induced current pulse achieves its maximum approximately when the maximum number of charge carriers are being collected. If the difference between the initial interaction position and the effective interaction position is large, this will affect the position of the peak of the induced current pulse and therefore the time difference between the front-side and back-side pulses as estimated in this work. For interactions of 70 keV, due to the initial track length, there will be a larger variation among the estimated time differences for each interaction position compared to the case with interactions of 10 keV.

It is expected that electronic noise will increase the variation among the estimated time differences for the each interaction position further as it affects the precision of the fit performed to estimate the time of each pulse. In the case with noise corresponding to a standard deviation of σ=0.05  keV [Figs. 7(b) and 7(e)], the time differences are very similar to the ideal case with no electronic noise. The standard deviations increase slightly for the 10-keV case but are approximately the same in the 70-keV case. With a noise level of σ=0.5  keV [Figs. 7(c) and 7(f)], the average time differences remain very similar to the ideal case and the σ=0.05  keV case. However, for the 10-keV case, the standard deviations increase drastically and become higher than for the 70-keV case due to the decreased SNR. This indicates that at 70 keV, the effect of the initial charge carrier distribution is larger than the effect of noise for the evaluated cases.

Apart from the initial charge distribution, the appearance of the induced current pulses is also affected by diffusion. Diffusion increases the size of each charge distribution over time and therefore also affects the width of the induced current pulses. In general, the further the charge carriers have to drift before being collected, the larger the charge cloud will be upon collection. With the presented method of estimating the time of the pulse peak, the charge cloud size upon collection affects the slope of the integrated current pulse. In the case of a very wide pulse, it can be expected that the precision of estimating the time of each pulse as proposed in this work increases due to having more data points in the slope of the curve. On the other hand, with a large charge cloud it is also expected that the amount of charge sharing increases. This means that the fraction of charge that is being collected by the electrode pair that is used to estimate the time of each pulse decreases. In a case with electronic noise, this will decrease the performance of estimating the time of each pulse due to decreased SNR. Overall, the increase in charge cloud size due to diffusion can be governed using the bias voltage. Since the bias voltage affects the electric field in the detector, increasing the bias voltage reduces the time required for the charge carriers to be collected. In this work, a bias voltage of 200 V was used in alignment with our previous publications.^8^^,^^9^ However, this is not necessarily the optimum for maximizing the performance of estimating the time of each pulse.

In Fig. 8, the effect of the interaction position in the x direction can be seen for the 10-keV case [(a)–(c)] and 70-keV case [(d)–(f)], respectively. For all presented noise cases and energies, it is clear that the average time differences have the same appearance. For the 10-keV case, the standard deviations (indicated by the error bars) increase significantly when including noise corresponding to a standard deviation of σ=0.5  keV. In the case of interactions on the pixel edge, this effect is enhanced due to charge sharing, in which the deposited energy is split between the investigated pixel and the neighboring pixel, which decreases the SNR. For the 70-keV case [Figs. 8(d)–8(f)], there is very little effect of both the noise level and the interaction position in the x direction.

The standard deviation for each interaction position and noise assumption is presented in Fig. 9 and shows a large difference between the 10- and 70-keV cases. For the 10-keV case [Fig. 9(a)], the standard deviations are low for the assumed ideal detector with no noise and for the noise case with σ=0.05  keV. Interaction positions on the pixel edge have a higher standard deviation for all three noise cases. The largest standard deviations are obtained for the noise case with σ=0.5  keV for which interactions on the pixel edge exhibit the largest standard deviations overall. For interactions in the middle of the pixel, the standard deviation is the largest for interaction positions close to the back side of the detector. For such positions, the required drift time for the holes to reach the front side electrode is long and the charge carriers therefore diffuse a lot. Many holes therefore become collected by the neighboring electrodes which reduces the total energy detected by the investigated pixel. This reduces the SNR which has a negative effect on estimating the time of the interaction. The largest standard deviations are obtained for interactions at the pixel edge. For such cases, the SNR is at its minimum.

The standard deviations for the 70-keV case [Fig. 9(b)] exhibit fewer differences compared to the 10-keV case. Regardless of noise level and interaction position in the x direction, the standard deviations show a similar behavior: the standard deviation increases with an increasing distance to the front-side electrode. However, for the position closest to the back side (90%), the standard deviation is slightly lower compared to the position 80% from the front-side electrode. For this interaction position, the time estimation for the back-side pulse has a smaller standard deviation compared to other interaction positions. This propagates to the time difference between the front-side and back-side pulses and results in a lower standard deviation.

Regarding time resolution, the charge collection times presented in Fig. 10 show a similar appearance compared to the time difference as a function of the interaction position. For the 10-keV interactions, the effective interaction position, corresponding to the distribution of the released charge carriers, typically corresponds well to the actual interaction position. For this interaction energy, the average electron track length was 1  μm. Since the interactions were simulated in nine different positions in the y direction, the scatter plots show nine distinct clusters for the 10-keV data. For 70-keV interactions, on the other hand, the average electron track length was 28  μm and the effective interaction position did not necessarily coincide with the actual interaction position. Since the estimated time of the front-side and back-side pulses approximately correspond to the time in which most charge carriers are collected, the charge collection time, therefore, varies with the difference between the actual interaction position and the effective interaction position. For 70-keV interactions, this reduces the performance of estimating the interaction position, at least if not some more advanced signal processing is developed that can account for the track of the photoelectron. However, this does not necessarily reduce the performance of estimating the time of the interaction. An indication of this can be seen in Fig. 10(a), for which the 70-keV data show a distinct line with no true outliers. In Fig. 10(c), due to the decrease in SNR that comes with an increased noise level, estimating the time of each pulse increases in difficulty, which affects the time difference between the front-side and back-side pulses. This is especially true for interactions of 10 keV.

The effect of the interaction position in the x direction, presented in Fig. 11, shows that the results for interactions on the pixel edge are similar to those for interactions in the pixel middle. The main effect for interactions on the pixel edge is that the same charge collection time is achieved at a slightly higher time difference compared to the case with interactions at the pixel middle. This occurs since the distance to the electrodes is slightly larger for charge carriers at the pixel edge compared to charge carriers at the pixel middle. For the 0-keV case, Figs. 11(a)–(c), the charge collection times appear as clusters for the ideal and the σ=0.05-keV cases. With a noise level of σ=0.5  keV, the charge collection time varies a lot for the same value of the time difference. This is especially true for interactions on the pixel edge for which the data show that the same time difference can be achieved for a range of charge collection times that is ∼3  ns wide. In contrast, the corresponding interval is ∼2  ns for interactions at the pixel middle. In general, the large variation in charge collection time results from the reduced SNR which, for interactions on the pixel edge, is further reduced due to charge sharing. For the 70-keV case [Figs. 11(d)–11(f)], the fitted curves are very similar compared to the 10-keV ideal and σ=0.05-keV cases. However, the 70-keV scatter plots show a larger variation of the charge carrier collection time for each value of the time difference. This is mainly caused by the initial distribution of the charge carriers. For the σ=0.5-keV case, the behavior is similar in comparison to the lower noise cases, which indicates that estimating the time of high-energy interactions is robust with respect to the electronic noise level.

The summarized results in Table 1 show that the highest timing resolution is achieved for the ideal case based on interactions of 10 keV and corresponds to ∼10  ps. For the 10-keV case in general, the performance decreases for interaction positions on the pixel edge. Based on the combined data, the timing resolution ranges between ∼90  ps and 1 ns depending on the noise level. For interactions of 70 keV, similar performance is obtained for both the ideal and the σ=0.05-keV case. For the combined data, the performance at σ=0.05-keV results in a time resolution that is ∼30% higher than for the corresponding case with 10-keV interactions. At a noise level of σ=0.5  keV, the time resolution is ∼130  ps, which results in a time resolution that is similar to the σ=0.05-keV case with interactions of 10 keV. From this table, it can be concluded that the time resolution is highest for low-energy interactions in the case of an ideal detector with no noise. However, for a realistic detector with electronic noise, our results indicate that the highest time resolution is achieved for high-energy interactions due to the higher SNR. Overall, our results indicate that the time of the interaction can be determined with an accuracy of at least 1 ns.

In this work, only one set of electrodes was utilized. However, we expect the performance of the presented method to increase if the number of included electrodes is increased. This is especially true for cases in which there is a lot of charge sharing, i.e., for interactions on the pixel edge. For a case in which data are extracted from a larger number of electrodes, the curve fit performed for electrodes on each side of the detector could be synchronized so that the optimal curve fit to data from all electrodes on the same side of the detector is achieved. In the case of 10-keV interactions occurring close to, or on the pixel edge, we expect this to improve the performance. However, for more central interaction positions, the SNR might be too low in the neighboring electrodes, which reduces the benefit of including more electrodes when estimating the time of the pulses.

The template curves that were used in this work to estimate the time of the front-side and back-side pulses were calculated for each interaction position. However, in a realistic detector implementation, it might be desirable to use a single-template pulse. For future work, it is therefore of interest to evaluate the effect of the template pulse shape. Although this is yet to be evaluated, we expect the performance to be similar even with a single template pulse. Due to diffusion, the charge clouds are expected to have roughly the same distribution upon collection, although the charge clouds may vary in size depending on the interaction position. Based on this, the induced currents can vary in duration but should consist of the same shape [see Fig. 6(a)]. Since the curve fit in this work is performed based on three parameters: the curve amplitude, the curve slope, and the position of the curve in time, as long as the template pulse corresponds to a signal with the same symmetry as the induced current pulses, it should be possible to alter the curve slope in order to account for the width of the pulse. However, regarding interaction positions in the x direction, we have previously presented a method to obtain 1-μm resolution for low-energy interactions which, if desired, could enable using different template curves based on the interaction position in the x direction.^9^

The count-rate performance of the presented detector geometry is expected to be high. We have previously presented detector prototypes with a count-rate performance that is linear up to $90\;\mathrm{Mcps}/{\mathrm{mm}}^{2}$.^25^ The performance is then limited by the dead time of the system, which relates to the pulse length that is associated with the electronic pulse from the readout electronics. For that prototype system, the reported dead time is 110 ns. In this work, the signal is extracted at the CSA output, where the signal is 10 times shorter than this, as can be seen in Fig. 6. Assuming the same detector geometry as in the referenced work, this results in a count-rate linearity of up to $900\;\mathrm{Mcps}/{\mathrm{mm}}^{2}$. For CT examinations, it has been shown that the maximum photon fluence ranges between 300 and $600\;\mathrm{Mcps}/{\mathrm{mm}}^{2}$.^26^ It should further be noted that the detector assumed in this work assumes a much smaller pixel size which will further improve the count-rate capability.

The required timing resolution to resolve the interactions corresponding to a single incident photon was estimated in our previous work regarding Compton coincidence detection.^8^ Based on a $25\times 25\;{\mathrm{mm}}^{2}$ detector area as seen from the x-ray source, it was concluded that a time window of ∼1  ps was required given an incident photon flux of $340\;\mathrm{Mcps}/{\mathrm{mm}}^{2}$, corresponding to the maximum CT flux in an intermediate-sized chest image.^26^ At a more clinically relevant incident photon flux of $10\;\mathrm{Mcps}/{\mathrm{mm}}^{2}$, the corresponding time window is 0.16 ns. Correspondingly, allowing five photons per time window, the required time resolution becomes ∼6  ps at $340\;\mathrm{Mcps}/{\mathrm{mm}}^{2}$ and 0.8 ns at $10\;\mathrm{Mcps}/{\mathrm{mm}}^{2}$. For the latter case, the required timing resolution aligns with the timing resolution presented in this work and we, therefore, see the potential of applying the proposed method and detector configuration for coincidence detection.

Current silicon detectors for photon-counting CT do not utilize timing information. The photon-counting logic is instead based on sampling the processed signal from the silicon diode according to a clock cycle of a few nanoseconds. Whenever the processed signal exceeds a lowest threshold, a count is registered and the signal amplitude is sampled for a set number of clock cycles, typically around 100 ns. However, in a single-sided silicon strip detector, it has previously been shown that a timing resolution corresponding to an RMS error of 1.5 ns can be achieved.^27^ The result is then based on the movement of holes solely in comparison to electrons and holes as included in this work. Further, for applications in high-energy physics, silicon strip detectors with a time resolution of between 34 and 16 ps have been presented.^28^

Overall, our results indicate that it is possible to improve the time resolution in silicon detectors with double-sided readout, especially for high-energy interactions. However, the presented method can, as indicated by the results, also be used to improve the spatial resolution. By improving the time resolution, our long-term goal is to resolve Compton interactions to identify interactions that originate from the same incident photon. This could enable reducing the amount of tungsten shielding used to absorb photons that scatter within the detector and thereby improve the dose efficiency by increasing the energy resolution and the effective area of the detector. There could also be potential in applying the presented methodology to eliminate object scatter based on time information.^29^^–^^31^ However, this requires a pulsed x-ray source, which is not available in the clinic today. Further, we also see potential future applications in time-of-flight PET, which is a field that currently revolves heavily around scintillation detector technology.^32^

For future work, it is desirable to evaluate the presented methodology for a wide incident spectrum, compared to the two monoenergies used in this work. Further, it is also of interest to design and manufacture prototype detectors with double-sided readout. Regarding the readout electronics, the implementation is still unknown. However, although the required sample rate is yet to be determined, analog-to-digital converters with a sampling rate of 20  GS/s, as assumed in this work, have previously been reported.^33^^,^^34^ To reduce complexity of a first prototype system, it would be desirable to extract the sampled signals from the detector and perform the time estimation offline.

## 5 Conclusion

We have presented a method for time of interaction measurements in deep silicon detectors for photon-counting spectral CT. We use a double-sided readout with electrodes positioned on both sides of the silicon wafer. Based on the induced signals on each side of the sensor, it is possible to infer the time of the interaction. Our results show that a time resolution of ∼0.1  ns can be achieved for a case with a very low electronic noise level. Assuming a higher noise, corresponding to a lower power consumption, the time resolution is ∼1  ns. The presented timing resolutions show potential for enabling separating single photon interactions in order to estimate the incident photon energy and thereby improving the spectral resolution of the detector. Future work involves evaluating the effect of including multiple pairs of opposing electrodes as well as the effect of the proposed method on image quality and dose efficiency.
